# Comparison of real-time feedback and debriefing by video recording on basic life support skill in nursing students

**DOI:** 10.1186/s12909-022-03951-1

**Published:** 2023-01-25

**Authors:** Mohammad Sajjad Ghaderi, Javad Malekzadeh, Seyedreza Mazloum, Tayebe Pourghaznein

**Affiliations:** 1 Department of Nursing, Torbat Jam Faculty of Medical Sciences, Torbat Jam, Razavi Khorasan Province Iran; 2 Clinical Research Development Unit, Sajjadieh Hospital, Torbat Jam Faculty of Medical Sciences, Torbat Jam, Razavi Khorasan Province Iran; 3 Nursing and Midwifery Care Research Center, Mashhad University of Medical Sciences, Mashhad, Iran

**Keywords:** Real-time feedback, Debriefing by video recording, Skill, Basic life support, Nursing students

## Abstract

**Background:**

Cardiopulmonary resuscitation skill have a direct impact on its success rate. Choosing the right method to acquire this skill can lead to effective performance. This investigation was conducted to compare the effect of Real-time feedback and debriefing by video recording on basic life support skill in nursing students.

**Methods:**

This quasi-experimental study was performed on 67 first year nursing students. First, a theoretical basic life support (BLS) training session was held for the all participants, at the end of session the pre-test was taken. Students were randomly assigned to two groups. A 4-hour practical BLS training session was conducted in the real - time feedback group as well as the debriefing by video recording group, and at the end of the training, a post-test was taken from each group. Each group received a post-test. Data were analyzed using SPSS 25 software.

**Results:**

Results showed a significant difference between mean (SD) of debriefing by video recording group in pre-test and post-test (*p* < 0.001) and in the real-time feedback group there was a significant difference between mean (SD) in pre-test and post-test (*p* < 0.001), respectively. In addition, there was no significant difference between the mean score of basic life support skill in real-time feedback and debriefing by video recording.

**Conclusions:**

Both real-time feedback and debriefing by video recording were effective on basic life support skill.

## Introduction

The burden of cardiovascular disease and the sudden increase in the number of cardiac arrests is one of the most important health problems in the world, which imposes high costs on the health care system of countries every year [1]. An estimated 400,000 persons in the United States and 700,000 persons in Europe die every year due to the lack of cardiopulmonary resuscitation (CPR) in critical time [2]. The American Heart Association and European Resuscitation Council guidelines (2020–2021) stated that high-quality chest compression is the key to success in the adults’ chain of survival. To increase the chance of victim’s survival and resuscitation performance, chest compression depth of 6–5 cm, a rate between 100 and 120 compressions per minute, allowing chest recoil and reducing CPR interruptions (less than 10 seconds) are recommended [3, 4]. Despite the importance of this issue, and CPR guidelines and rescuers training, resuscitations of not always meet recommended standards [5].

Resuscitation education is a necessary element of skills training for nurses who are likely to be first-line rescuer because they spend significant time alongside patients who are experiencing in-hospital cardiac arrest [6]. Thus, it would be beneficial for Undergraduate medical sciences students to have proper knowledge and performance about CPR to strengthen their skills for future use [7].

Lack of feedback to rescuers is one of the barriers to high-quality CPR [8]. With the advancement of technology in the field of cardiopulmonary resuscitation, studies have shown the effectiveness of feedback devices in improving resuscitation performance and increasing victim’s survival [9–11]. On the other hand, studies have shown the effect of using the debriefing training method on the performance of rescuers [5, 12–14].

The necessity of continuous and up-to-date training is increasingly felt in the survival of victims. In this regard, finding a training method with the highest effect which can also retain the learning of rescuers is of particular importance.

Therefore, researchers performed the present study aiming to compare of real-time feedback and Debriefing by Video Recording on basic life support skill in nursing students.

## Methods

This study was quasi-experimental (pretest-posttest). Research sample in this study included 67 first year nursing students studying in Mashhad School of Nursing and Midwifery in the academic year 2019–2020 who were selected by convenience sampling method. Inclusion criteria were: no clinical work experience, no participation in CPR training courses and willingness to participate in the study. Exclusion criteria were Persons who attended a training session or similar study at the same time and those who did not want to continue working from the study. In this study, the sample size was calculated (*n* = 74) using the formula for comparing means, 95% confidence level, 80% test power and citing the study of Agbayani et al. [15].

## Measurement and instrument

The instruments used in the research were demographic information questionnaire, Kolb learning style inventory and BLS checklist with 18 items. This researcher-made checklist was prepared after reviewing samples of similar foreign and domestic checklists and based on the latest changes in the clinical guidelines of the American Heart Association and European Resuscitation Council (2020–2021). Scoring consisted of not doing at all (zero) and doing completely and correctly (one), and the score range considered for this observation checklist was from 0 to 14 with four items for CPR meter 2 laerdal (including: accuracy percentage of chest compression depth, accuracy percentage of chest compression rate, accuracy percentage of complete chest recoil and accuracy percentage of chest compression fraction) were evaluated to assess the performed chest compression. Content validity was assessed for tool validity so that the study tool was provided to 9 faculty members of Mashhad School of Nursing and Midwifery and it was confirmed by coefficient of variation ratio (CVR): 0.99 and content validity index (CVI): 1. The reliability of the basic life support skill checklist of rescuers was approved using Richardson’s Koder method with a coefficient of 0.804. The reliability of the Kolb Learning Style Inventory was confirmed with a Cronbach’s alpha coefficient of 0.92.

## Intervention

**Fig. 1:**
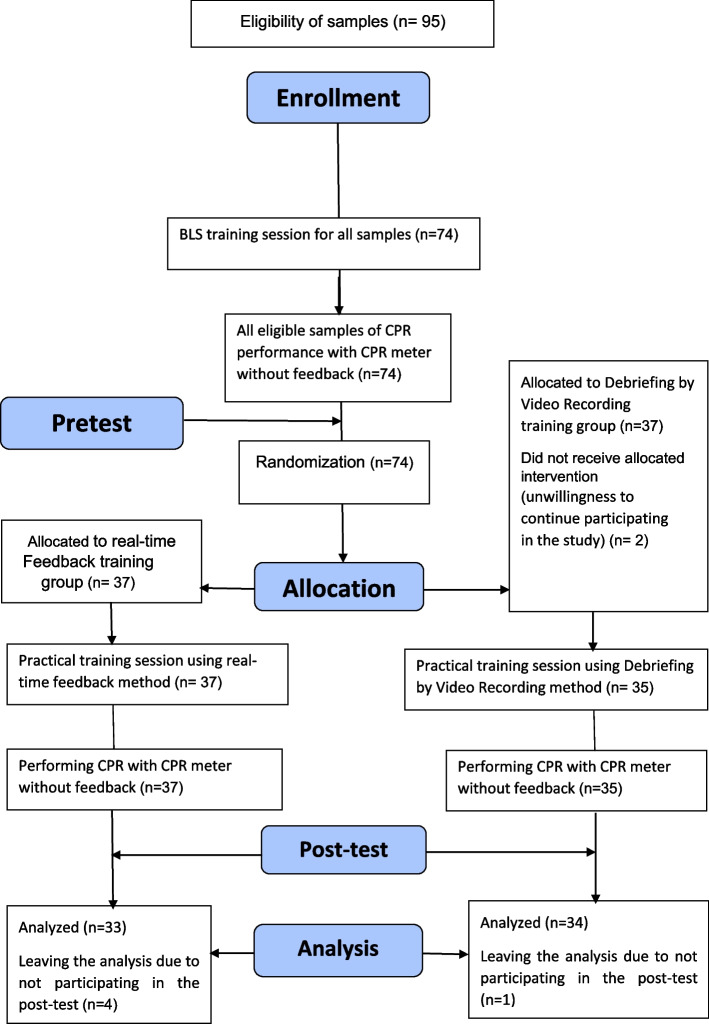
Study Method Diagram

After obtaining permission from the ethics committee of Mashhad University of Medical Sciences (MUMS) and approval of the Nursing and Midwifery Education Department, students’ informed written consent was obtained. The participants were included in the study in an accessible manner according to the inclusion criteria. First, a theoretical BLS training session (by the second author) was held for all students for 4 hours and pre-test was taken at the end of the session. Participants were assigned to two groups of real-time feedback and debriefing by video recording using a random sequence generated using SPSS software, with 37 participants in each group. For the practical BLS training session, each group was divided into smaller groups of 5–6 persons. In the group of real-time feedback and debriefing by video recording, the students’ practical BLS training session was held for 4 hours as a training using a manikin. In the real-time feedback group, the resuscitation steps and how to provide feedback of the device were practically taught. Next, cardiopulmonary resuscitation was performed and the performance of rescuer was corrected using the feedback of the device. In the Debriefing by Video Recording group, first the practical BLS training of resuscitation steps was provided and the students performed resuscitation operations on the manikin and their performance was filmed. The recorded videos were then reviewed so that students could improve their BLS skill again. At the end of the session, students in both groups were retested (Fig. 1).

## Data analysis

The collected data were analyzed using SPSS software version 25. Kolmogorov-Smirnov and Shapiro Wilk tests were used to determine the normal distribution of data. Mann-Whitney test was used to compare the mean basic life support skill between the two groups of real-time feedback and debriefing by video recording before and after the intervention. Paired t-test was also used to compare the means in both groups before and after the intervention.

## Results

Out of 74 students participating in the study, 7 were excluded from the study. The total age range of students was from 18 to 30 years. The majority of participants in this study were female (61.1%). The results of Chi-square test showed that the frequency distribution of sex (*P* = 0.598) and semester (*P* = 0.318) were not significantly different between the two groups. Mann-Whitney test also showed that there was no significant difference in the frequency distribution of age between the two groups (*P* = 0.250) (Table 1). The results of analysis of variance (ANOVA) test showed a significant relationship between basic life support skill score and learning styles (df = 63.3, F = 2.9, *P* = 0.038). The post hoc test showed this difference between absorption and adaptive learning style, as well as divergent with adaptive learning style.

**Table 1:** Comparison of the frequency distribution of demographic characteristics in students of the two groups of real-time feedback and debriefing by video recording

		Real time feedback (*n* = 33)	Video debriefing (*n* = 34)	*p*-value*
Age Mean (SD)		20.2(30.46)	19.2(79.15)	0.250
Sex (%)	Female	39.4	38.2	0.598
	Male	60.6	61.8	
Academic semester (%)	First semester	57.6	67.6	0.318
	Second semester	42.4	32.4	

In the pre-test stage, the mean basic life support skill was 6.4 ± 1.7. The lowest score was 3 and the highest score was 10. The results of Mann-Whitney test showed that there was a statistically significant difference (*P* = 0.009) between the two groups of real-time feedback and debriefing by video recording in the pre-test stage in terms of mean BLS skill. Due to the lack of homogeneity of the pre-test stage, analysis of covariance was used to remove its effect. The results showed that the mean basic life support skill of the post-test stage did not differ significantly (*P* = 0.341) between the two groups. The mean modified post-test score was 11.69 ± 1.18 in the real-time feedback group and it was 12.11 ± 1.09 in the debriefing by video recording group. The mean score of basic life support skill in post-test stage was 11.91 ± 1.15 with the lowest score of 9 and the highest score of 14. The results of Mann-Whitney test showed that there was no statistically significant difference (*P* = 0.136) between the two groups of real-time feedback and Debriefing by Video Recording in the post-test stage. In the intragroup comparison, the results of paired t-test showed that in the real-time feedback group (*P* < 0.001) and in the Debriefing by Video Recording group (P < 0.001), the mean score of pre-test and post-test skill was statistically significant (Table 2). Since the factors evaluated by CPR meter 2 are objective and represent standard chest compression, 4 items of chest compression depth, chest compression rate, chest recoil and ratio of chest compression time to total cardiopulmonary resuscitation time are presented in a separate table (Table 2).

**Table 2:** Mean and standard deviation of basic life support skill of nursing students studied in two groups of real-time feedback and debriefing by video recording

		Real time feedback(n = 33)	Video debriefing (n = 34)	Z	*P*-value*	effect size
CPR performance score (Mean (SD))	Pre-training	5.81(1.53)	7.02(1.83)	−2.617	0.009****	
	Post-training	11.69(1.18)	12.11(1.09)	−1.492	0.136	
	*P*-value***	*P* < 0.001	*P* < 0.001			
CC Depth (%)	Pre-training	15.30(27.72)	18.85(33.76)	−0.870	0.384	
		3(11.5IQR)	0(18IQR)			
	Post-training	76.66(22.65)	49.05(37.22)	−2.975	0.003	0.79
		87(31IQR)	44.5(76IQR)			
	*P*-value**	*P* < 0.001	*P* < 0.001			
CC Rate (%)	Pre-training	11.48(20.54)	11.97(25.24)	−0.069	0.945	
		0(14.5IQR)	0(6IQR)			
	Post-training	60.54(19.60)	48.08(32.04)	−1.480	0.139	0.39
		61(25.5IQR)	52.5(55.5IQR)			
	*P*-value**	*P* < 0.001	*P* < 0.001			
CC Recoil (%)	Pre-training	83.06(30.19)	83.58(29.96)	−0.171	0.864	
		100(16.5IQR)	100(19.5IQR)			
	Post-training	78.06(19.65)	71.52(25.86)	−0.672	0.502	0.31
		84(30IQR)	77.5(40.5IQR)			
	*P*-value**	0.161	0.033			
CC Fraction (%)	Pre-training	78.66(26.03)	83.82(19.70)	−0.628	0.530	
		93.5(45.25IQR)	95.5(30.25IQR)			
	Post-training	70.18(11.11)	61.91(12.21)	−2.722	0.006	0.7
		68(12 IQR)	63.5(17.25IQR)			
	*P*-value**	0.064	< 0.001			

*Mann–Whitney U **Wilcoxon signed-rank test

*** T paired test

Values are presented as median (interquartile range) or mean (SD) (95% confidence interval)

*P* < 0.05, statistically significant

****: In the pre-test stage, it did not become significant by deleting the effect (*P* = 0.341)

Chest Compression Depth: According to Mann-Whitney test, there was no statistically significant difference between the two groups of real-time feedback and Debriefing by Video Recording in terms of the average percentage of the accuracy of chest compression depth performed in the standard range in the pre-test stage (*P* = 0.446), but in the post-test stage, there was a statistically significant difference (*P* = 0.003). In intragroup comparison, the results of Wilcoxon test showed that in both groups of real-time feedback and debriefing, the mean percentage of the accuracy of chest compression depth in the standard range before and after the intervention had a statistically significant difference (P < 0.001) (Table 2).

Chest compression rate: Based on Mann-Whitney test, there was no statistically significant difference between the two groups of real-time feedback and Debriefing by Video Recording in terms of the average percentage of the accuracy rate of chest compression performed in the standard range in pre-test (*P* = 0.0925) and post-test (*P* = 0.191). In the intragroup comparison, the results of Wilcoxon test showed that in both groups, there was a statistically significant difference (*P* = 0.001) in the mean percentage of the accuracy rate of chest compression performed within the standard range in the pre-test and post-test stages (Table 2).

Chest compression recoil: According to the Mann-Whitney test, there was no statistically significant difference between the two groups of real-time feedback and Debriefing by Video Recording in terms of the average percentage of chest recoil in the pre-test (*P* = 0.744) and post-test (*P* = 0.404) (Table 2). In the intragroup comparison, the result of Wilcoxon test showed that in the real-time feedback group, the mean percentage of the accuracy of chest recoil of the chest compression performed before and after the intervention was not statistically significant (*P* = 0.161). However, the result of Wilcoxon test for the Debriefing by Video Recording group showed a significant difference (*P* = 0.033) (Table 2).

Chest compression fraction: Based on Mann-Whitney test, there was no statistical difference between the two groups of real-time feedback and Debriefing by Video Recording in terms of the average percentage of the accuracy rate of chest compression performed in the standard range in the pre-test stage (*P* = 0.519), however, there was a statistically significant difference (*P* = 0.006) in the post-test stage. In the intragroup comparison, the results of Wilcoxon test showed that in the real-time feedback group, regarding the mean percentage of the accuracy of chest compression fraction before and after the intervention, there was no statistically significant difference (*P* = 0.064). Nevertheless, the results of this test for Debriefing by Video Recording group had a significant difference (*P* < 0.001) (Table 2).

## Discussion

Findings of the study showed that basic life support skill significantly increased due to the application of both real-time feedback training and Debriefing by Video Recording methods, while there was no significant difference between the two training methods.

Regarding the effect of real-time feedback method, our study findings were in line with the findings of Tanaka et al. study, which examined the effect of real-time auditory feedback device on the skill of non-professionals trained in Japan. Their results showed that the use of feedback devices in cardiopulmonary resuscitation has been able to significantly improve quality of CPR [10]. The results of Brown et al., which examined the effect of using CPR feedback device on healthcare provider workload during simulated cardiac arrest, showed that using CPR feedback device after exercise effectively increased the skill of rescuers which is consistent with the results of our study [16]. The use of auditory and visual feedback devices during cardiopulmonary resuscitation improves resuscitation skill [9]. On the other hand, studies have been conducted on real-time feedback devices that are not in line with the findings of our study, such as the study of Zapletal et al. which was not in line with the results of our study. This study compared the effect of three CPR feedback devices and the standard BLS method, in the results showed that one of the real-time feedback devices did not have a significant effect on the skill score and like the standard method, chest compression was performed on the manikin [17]. Among the causes of inconsistency with our study, the use of three different devices and shorter intervention time could be considered. The findings of the present study were not in line with the study of Kramer-Johansen et al. which the results showed that auditory feedback devices were not effective in quality of cardiopulmonary resuscitation [18]. Studies on the impact of different types of real-time feedback devices have shown that cardiopulmonary resuscitation parameters have been the main monitoring method for resuscitation feedback. In sudden cardiac arrest, when non-professional rescuers are initially present at the scene, high-quality cardiopulmonary resuscitation can increase the victim’s chances of survival [19, 20]. In the present study, Debriefing by Video Recording method was able to significantly improve the skill of rescuers. The findings of our study were consistent with the findings of the study of Aghajani et al. because the results of the study examined the effect of video feedback on the performance of non-professional rescuers, it showed that video feedback had a significant effect on students’ performance [15]. The results of some previous studies were also in line with the present study because they showed that the training method using debriefing sessions was effective on cardiopulmonary resuscitation skill [21–23]. In addition to these studies, we can refer to the results of the study Ostovar et al., which was not in line with our results and compared effects of debriefing methods on psychomotor skills, self-confidence, and satisfaction in novice nursing students. The findings of this study showed that there was no significant difference in the average CPR skill in the two training groups, but both methods were able to improve the skill significantly [13]. In our study, debriefing sessions were held after cardiopulmonary resuscitation. The recorded videos were reviewed and the students reviewed their strengths and weaknesses in cardiopulmonary resuscitation. Debriefing by Video Recording was much more effective than verbal reporting and it was more effective in learners’ improved performance [12]. Limitations of the present study included the implementation of these training methods only in one semester for nursing students, which the society was limited to students in this field. Hence, generalizing the results to other groups is less reliable.

Based on the findings of the present study, in both groups, the evaluation factors by CPRmeter 2 feedback device increased significantly after the intervention, but the rate of increase of these criteria was higher in the real-time feedback group than Debriefing by Video Recording group in the post-test. Intergroup comparison of real-time feedback method compared to Debriefing by Video Recording method in chest compression depth parameters (*P* = 0.003) and chest compression fraction (*P* = 0.006) were significantly increased. Participants in the real-time feedback group were able to adjust their chest compression more peacefully within the standard range of chest compression and device feedback, as the teacher did not interfere in providing feedback, but the participants in the debriefing by video recording group maybe because they were filmed during cardiopulmonary resuscitation, were doubly stressed, which reduced their ability to perform cardiopulmonary resuscitation, especially during the performance of the standard chest compression. Researchers suggest that the effect of real-time feedback training methods and debriefing by video recording on the retention of psychomotor cardiopulmonary resuscitation skill will be examined and analyzed in future research.

## Conclusions

Based on the findings of the present study, both real-time feedback and debriefing by video recording interventions were effective on improving CPR skill. Therefore, both methods are recommended as standard educational methods. Since the AHA recommendation according to the guideline in improving the quality of CPR, attention and emphasis is on 4 parameters (chest compression depth, chest compression rate, chest recoil and chest compression fraction) and CPR meter compared to debriefing by video recording method leads to further improvement of these parameters, training using CPR meter is recommended.

## Data Availability

All data generated or analyzed during this study are included in this published article.
